# Evaluation and Validation of Reference Genes for Gene Expression Analysis Using qRT-PCR in the Sugarcane Stem Borer *Chilo sacchariphagus* (Lepidoptera: Pyralidae)

**DOI:** 10.3390/insects15080594

**Published:** 2024-08-04

**Authors:** Zhixiong Wang, Xiankun Shang, Jili Wei, Xiaoli Tian, Yi Liu, Guohui Zhang

**Affiliations:** 1 College of Agriculture, Yangtze University, Jingzhou 434025, China; ulyanof@163.com (Z.W.); pangyu0963@163.com (Y.L.); 2 Key Laboratory of Sugarcane Biotechnology and Genetic Improvement (Guangxi), Ministry of Agriculture and Rural Affairs/Guangxi Key Laboratory of Sugarcane Genetic Improvement/Sugarcane Research Institute, Guangxi Academy of Agricultural Sciences, Nanning 530007, China; shanglei8289@163.com (X.S.); wjl2004-7919@163.com (J.W.); 3 College of Life Science, Yangtze University, Jingzhou 434025, China; lyacxiaoli@163.com

**Keywords:** *Chilo sacchariphagus*, reference genes, gene expression analysis, real-time quantitative PCR

## Abstract

**Simple Summary:**

Gene expression analysis by quantitative real-time reverse transcription polymerase chain reaction (qRT-PCR) can provide strong evidence for scientists to understand the molecular mechanisms underlying various physiological processes. Selecting appropriate reference genes under specific experimental conditions are prerequisites for achieving the accurate results of qRT-PCR. Here, the expression stability of seven reference genes was evaluated in *Chilo sacchariphagus*, a destructive pest of sugarcane, under different experimental conditions, encompassing tissues, temperatures, and sexes. The expression patterns of *C. sacchariphagus* pheromone binding protein 1 gene (*CsacPBP1*) across three different experimental conditions mentioned above were evaluated to verify the results. The findings of this study will lay an important foundation for the future study on functional gene expressions in the pest.

**Abstract:**

*Chilo sacchariphagus* (Lepidoptera: Pyralidae) is an economically important sugarcane pest. Although numerous studies were conducted on the physiological responses in *C. sacchariphagus*, little is known regarding the genes regulating these physiological processes. Gene expression analysis by qRT-PCR can offer a significant indication for functional gene studies. To our knowledge, the reference genes of *C. sacchariphagus* have not been screened or evaluated, which hinders the functional gene study. In the present study, the stability of seven reference genes (*β-ACT*, *GAPDH*, *BTF3*, *28S*, *RPL7*, *EF1α*, and *SDHA*) was evaluated in *C. sacchariphagus* under different experimental conditions, including tissues (antenna, head, thorax, abdomen, leg, and wing), temperatures (4 °C, 25 °C, and 37 °C) and sexes (male and female), through RefFinder, which integrates four algorithms (Normfinder, BestKeeper, ΔCt method, and geNorm). The findings suggested that the combination of *β-ACT* and *RPL7* is ideal to analyze gene expressions in different tissues and at distinct temperatures, and *EF1α* and *SDHA* were suitable reference genes for comparing gene expressions between sexes. Finally, the expression profiles of *CsacPBP1* gene were evaluated, and the outcomes further confirm the importance of selecting fitting reference genes for normalization of qRT-PCR data. This study represents the first kind in screening out suitable reference genes for gene expression analysis in *C. sacchariphagus*. Information from this study is poised to galvanize future inquiry into the gene expression of *C. sacchariphagus*, an economically important pest of sugarcane.

## 1. Introduction

The sugarcane stem borer *Chilo sacchariphagus* is a serious sugarcane (*Saccharum* spp.) pest, originally from Java and has been described in most sugarcane cultivation regions in Asia [1,2,3]. *C. sacchariphagus* spread to the southern African country Mozambique in 1999 through the South-West Indian Ocean islands and posed a high threat to Australia [2,4,5,6]. It damages internodes along the stalks and causes “dead-heart”. On Reunion Island, which is an overseas department of France, the infestations of *C. sacchariphagus* result in 25.9% loss in cane mass and 27.9% loss in sucrose [7]. In China, where the average infestation rate of *C. sacchariphagus* reaches 70% to 80% in individual sugarcane-growing areas, the “dead-heart” rate of sugarcane exceeds 50% [8]. These damages result in a substantial reduction in sugarcane yield and economic returns [9]. Currently, integrated pest management (IPM) systems for managing *C. sacchariphagus* include: (1) the application of chemical pesticides [10]; (2) biological control strategies, encompassing the use of parasitoids, predators, and entomopathogenic microorganisms [11,12,13,14]; (3) behavioral manipulation, including the use of pheromones and trap plants [1,15]; (4) the selection of resistant sugarcane varieties [16]; and (5) the regulation of cropping systems, such as intercropping. However, there is a lack of research on the genes regulating the physiological processes of *C. sacchariphagus*. Recently, we have identified a significant number of genes, including thermo-tolerance genes and chemosensory genes that are essential to insect physiological processes, from the head transcriptome data of *C. sacchariphagus*. A future aim of the research group is to perform targeted investigation on the expression of the mentioned genes, for which qRT-PCR is a highly suitable method. However, the reference genes of *C. sacchariphagus* have not been screened or evaluated, which makes the analysis of gene expression patterns hard to carry out.

Since quantitative real-time reverse transcription polymerase chain reaction (qRT-PCR) has such advantages as remarkable stability, high efficiency, and accurate quantification, it has been widely utilized in gene expression analysis in biological research [17,18,19,20]. For the purpose of accurately determining the expression level of the target gene, it is crucial to choose reference genes to normalize the variances attributable to different treatments, RNA quality, amplification efficiency, and cDNA synthesis [21,22]. The expression level of the ideal reference gene or reference gene set used for qRT-PCR is postulated to be stably expressed under complex experimental conditions [23]. Nevertheless, the commonly used reference genes in qPCR experiments, including *β-actin*, *glyceraldehyde-3-phosphate dehydrogenase*, and *ribosomal RNAs*, may vary significantly under some experimental conditions, as reported by literatures [24,25,26]. The qPCR data may be incorrectly interpreted if an unstable reference gene was used for normalization [27]. Moreover, the number of reference genes employed for data normalization can affect the results of gene expression level [21,28]. To summarize, selecting appropriate reference genes and determining the optimal quantity of reference genes according to specific experimental conditions are prerequisites for achieving accurate normalization.

The goal of the current investigation was to determine the optimal reference genes for qRT-PCR analysis of *C. sacchariphagus* under different experimental conditions, including tissues, temperatures, and sexes for normalization. The expression stability of seven commonly used reference genes, including *β-actin* (*β-ACT*), *glyceraldehyde-3-phosphate dehydrogenase* (*GAPDH*), *basic transcription factor 3* (*BTF3*), *28S ribosomal* (*28S*), *ribosomal protein L7* (*RPL7*), *elongation factor 1 alpha* (*EF1α*), and *succinate dehydrogenase complex subunit A* (*SDHA*), were measured using geNorm [21], Normfinder [29], BestKeeper [30], ΔCt method [31], and RefFinder [32]. The geNorm ranks the expression stability of each reference gene by calculating their expression stability value (M), judged by the criterion that the smaller the M value, the more stable the expression of the reference gene [21]. The NormFinder uses a model-based approach to estimate expression variation of each reference gene, with lower values for genes being more stable [29]. The BestKeeper tool employs raw data (Ct values) and PCR amplification efficiency to generate an index, with lower index scores indicating better stability [30]. The ΔCt approach relies on relative pair-wise comparisons, and the standard deviation (SD) is used to rank stability (a lower SD implies a more stable gene) [31]. RefFinder was used to completely analyze the gene expression stability acquired from the above four approaches and estimate the geometric mean, resulting in a comprehensive ranking index [32]. In addition, the expression patterns of *C. sacchariphagus* pheromone binding protein 1 gene (*CsacPBP1*) under three different experimental conditions mentioned above were evaluated to verify the results. The results of this research will facilitate improvement of accuracy of qRT-PCR analysis and lay an important foundation for the future study on functional gene expressions in *C. sacchariphagus*.

## 2. Materials and Methods

### 2.1. Insect Rearing and Experimental Conditions

The same batch of *C. sacchariphagus* pupae were obtained from a colony continuously reared at the Sugarcane Research Institute, Guangxi Academy of Agricultural Sciences, Nanning, Guangxi Province, China. The male and female pupae were separately placed in a Petri dish (diameter 9 cm) with four layers of gauze, and a dampened cotton was added in the periphery of the Petri dish to regulate moisture. The Petri dish with lid removed was placed in the center of a plastic insect-rearing box, measuring 18 cm in length, 12 cm in width, and 6.5 cm in height, and then transferred to the artificial climate incubator (Wuhan Ruihua Instrument & Equipment Co., Ltd., Wuhan, China) with 25 ± 1 °C, 75 ± 5% relative humidity, and 14L:10D photoperiod. The newly emerged male and female moths were placed in a new insect-rearing box and fed with 10% honey water. The *C. sacchariphagus* adults were treated as follows:

Adult tissues: The tissues were collected in three biological replicates, with each replicate containing 80 antennae, 30 heads without antennae, 30 thoraces, 30 abdomens, 30 legs, and 30 wings from 80 healthy 3-day-old moths, with 40 males and 40 females, respectively.

Temperature: Twenty (10 males and 10 females) 3-day-old moths were incubated under three temperature points, including 4 °C (cold), 25 °C (ambient temperature), and 37 °C (hot) for 0.5 h, respectively. Five live moths of each sex were randomly collected at each temperature point, and three biological replicates were prepared.

Sex: Five healthy 3-day-old male and female moths were sampled, respectively, as one biological replicate. Three biological replicates were prepared.

### 2.2. Selection of Reference Genes and Primer Design

Seven commonly used reference genes (β-actin, β-ACT; glyceraldehyde-3-phosphate dehydrogenase, GAPDH; basic transcription factor 3, BTF3; 28S ribosomal, 28S; ribosomal protein L7, RPL7; elongation factor 1 alpha, EF1α; and succinate dehydrogenase complex subunit A, SDHA) were selected as candidates from the head transcriptome data of *C. sacchariphagus*. The sequences of these genes are listed in Table S1.

Primer Premier (version 5.0, PREMIER Biosoft International, Palo Alto, CA, USA) was used to design the primers according to the qRT-PCR primer design principles with primer lengths of 15–22 bases and amplification product length greater than 80 bp and less than 200 bp. At least two pairs of primers for each candidate reference gene were designed. The amplification specificity of each primer pair was confirmed through 1% agarose gel electrophoresis and melt curve analysis. Detailed information regarding the primers used for qRT-PCR analysis can be found in Table 1.

### 2.3. Total RNA Isolation and First Strand cDNA Synthesis

Total RNAs were extracted by using MiniBEST Universal RNA Extraction Kit (TaKaRa, Dalian, China) according to the manufacturer’s instructions. The integrity of the RNA was assessed through 1% agarose gel electrophoresis. The concentration and purity RNA were determined utilizing a spectrophotometer, Nanodrop ONEC (Thermo Fisher Scientific, Waltham, MA, USA). A quantity of 1 μg of total RNA was used for the synthesis of first-strand cDNA using the PrimeScript RT reagent Kit with gDNA Eraser (prefect realtime) (TaKaRa, Dalian, China) according to the manufacturer’s instructions and stored at −20 °C until use.

### 2.4. Quantitative Real-Time PCR (qRT-PCR)

qRT-PCR experiment was performed using TB Green *Premix Ex Taq* II (Tli RNaseH Plus) (TaKaRa, Dalian, China) on CFX connect Real-Time PCR detection system (Bio-Rad Laboratories, Hercules, CA, USA). The reactions were 25 μL mixture containing 12.5 μL TB Green *Premix Ex Taq* II (Tli RNaseH Plus), 9.5 μL nuclease free water, 1 μL each primer (10 μM), and 1 μL cDNA template. The thermocycling program was 1 cycle of 95 °C for 30 s, followed by 40 cycles of 95 °C for 5 s, annealing for 30 s, and 72 °C for 30 s. After the reaction, a melting curve analysis from 65 °C to 95 °C was applied to all reactions to confirm the specificity of the amplified product. To ensure the accuracy of the qRT-PCR results, no-template controls were used to detect the presence of primer dimers or contamination. A series of 10-fold dilutions of cDNA template were used to create standard curves using the linear regression model [30,33]. The efficiencies (*E*) of corresponding primers were then calculated according to the following equation: *E* = (10^[−1/slope]^ − 1) × 100% [34]. For each sample, three biological replicates were prepared, with each biological replicate containing two technical replicates.

### 2.5. Evaluation of Reference Gene Expression Stability

The raw data of qRT-PCR were analyzed using Bio-Rad CFX Manager software (version 3.0, Bio-Rad Laboratories, Hercules, CA, USA), and the cycle threshold (Ct) value of each sample was determined automatically. In this study, differences in Ct values greater than 0.5 for technical replicates of each sample were considered outliers. We eliminate outliers by repeating the experiment. The stability of seven reference genes across different experimental conditions was evaluated using geNorm [21], Normfinder [29], BestKeeper [30], and ΔCt method [31] according to the manuals of the algorithms. Furthermore, geNorm calculated the pairwise variations V_n_/V_n+1_ between two sequential normalization factors (NF) and determined the optimal number of reference gene required for accurate normalization, a V_n_/V_n+1_ value below 0.15 suggests that an additional reference gene will not significantly improve normalization. Finally, the comprehensive ranking of seven reference genes across different experimental conditions was analyzed by the web-based tool RefFinder (https://blooge.cn/RefFinder/ accessed on 16 November 2023) [32]. The RefFinder combined the four algorithms mentioned above and calculated the geometric mean of the ranking for each gene.

### 2.6. Validation of Recommended Reference Genes

To assess the validity of the experimental outcomes, the transcription levels of *C. sacchariphagus* pheromone binding protein 1 gene (*CsacPBP1*) were analyzed across different experimental conditions (tissues, temperatures, and sexes). The relative expression level of *CsacPBP1* was normalized using the most stable reference gene, the least stable reference gene, and the recommended combination of reference genes evaluated by RefFinder, respectively. Relative expression level of *CsacPBP1* were calculated using the 2^−ΔΔCt^ method [35]. Student’s *t*-test was used to compare the *CsacPBP1* relative expression levels normalized by two sets of reference genes, including separately normalized by the recommended combination of reference genes and the least stable reference gene or separately normalized by the most stable and the least stable reference gene, with a significance level set at *p* = 0.05.

## 3. Results

### 3.1. Specificity and Efficiency of Primers

**Figure 1:**
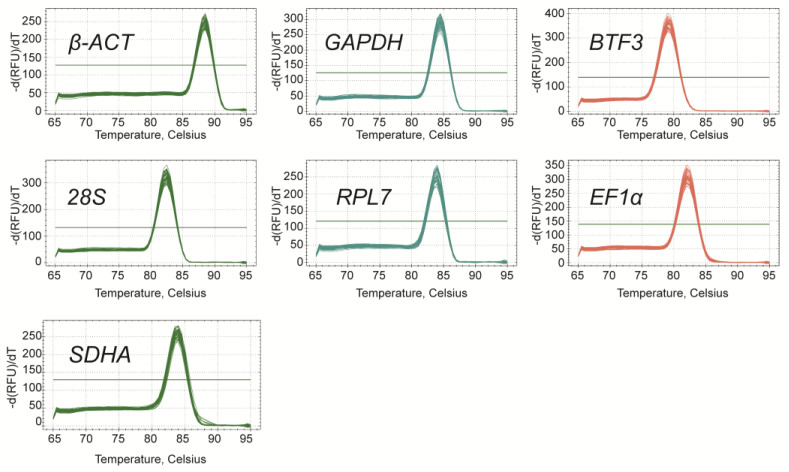
Melt curve analysis of seven reference genes. Abbreviation: β-ACT, beta-actin; GAPDH, glyceraldehyde-3-phosphate dehydrogenase; BTF3, basic transcription factor 3; 28S, 28S ribosomal; RPL7, ribosomal protein L7; EF1α, elongation factor 1 alpha; SDHA, succinate dehydrogenase complex subunit A.

The primer specificity of seven reference genes for qRT-PCR was verified by 1% agarose gel electrophoresis, and the single product with the expected size of each primer pair was amplified (Figure S1). A single peak for each primer pair was obtained in the melt curve analysis, which demonstrated that each primer pair amplified a unique product (Figure 1). Furthermore, the amplification efficiency (*E*) of seven primer pairs were between 90.02% (*28S*) and 107.87% (*EF1α*), and the correlation coefficients (*R*^2^) varied from 0.9922 to 0.9995 (Table 1). These results indicate that the specificity and efficiency of primers met the experimental requirements of qRT-PCR.

### 3.2. Expression Levels of Candidate Reference Genes

**Figure 2:**
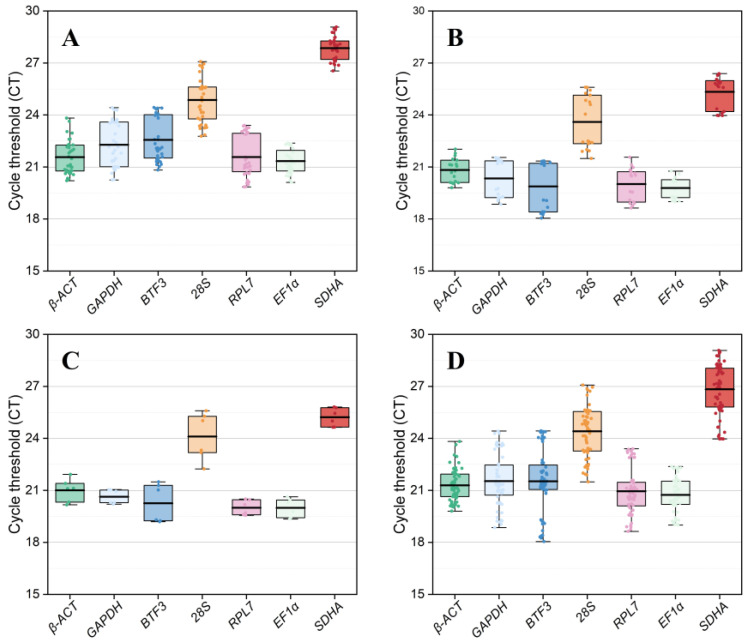
Expression levels of seven reference genes in *C. sacchariphagus*. The expression levels of reference genes are shown in terms of the mean Ct values in different treatments: (**A**) different adult tissues; (**B**) different temperature treatments; (**C**) different sexes; (**D**) all samples. Each box indicates 25th and 75th percentiles; the line across the box represents the mean. The abbreviations are listed in the description of Figure 1.

The expression levels of seven reference genes across three experimental conditions are shown in terms of the Ct values. Across all three experimental conditions, the average Ct values of seven reference genes ranged from 20.74 (*EF1α*) to 26.83 (*SDHA*), and the lowest and highest Ct values were 18.05 (*BTF3*) and 29.08 (*SDHA*), respectively (Figure 2D). Across different tissues, *SDHA* and *EF1α* had lower gene expression variations, whereas *28S* and *GAPDH* had higher (Figure 2A). Across different temperature treatments, the expression fluctuations were lower in *EF1α* and *β-ACT* and higher in *28S* and *BTF3* (Figure 2B). In males and females, variations in the expression levels were low in all candidate reference genes except for *28S* and *BTF3* (Figure 2C). A combination of the aforementioned results demonstrated that the expression fluctuations in *EF1α* and *β-ACT* were smaller, whereas the variations in *BTF3* and *28S* were higher (Figure 2D).

### 3.3. Expression Stability of Seven Reference Genes Across Different Tissues

**Figure 3:**
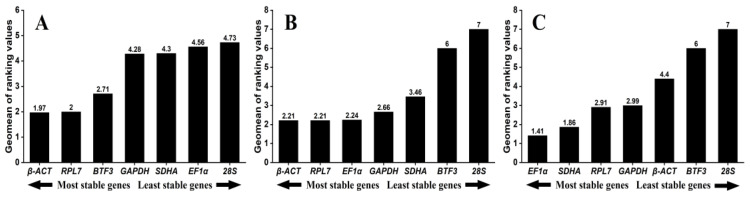
Expression stability of the seven reference genes in different samples of *C. sacchariphagus.* The stability of the reference genes was calculated using the Geomean method of RefFinder. A lower Geomean of ranking value indicates more stable expression. (**A**) different adult tissues, (**B**) different temperature treatments, (**C**) different sexes. The abbreviations are listed in the description of Figure 1.

**Figure 4:**
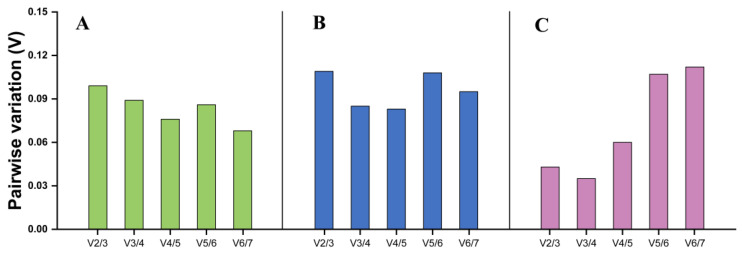
Optimal number of reference genes for data normalization in *C. sacchariphagus.* (**A**) different adult tissues, (**B**) different temperature treatments, (**C**) different sexes. The pairwise variations (V_n_/V_n+1_) were assessed by the geNorm program between normalization factors NF_n_ and NF_n+1_ to determine the optimal number of reference genes required for accurate normalization. A V_n_/V_n+1_ value below 0.15 suggests that an additional reference gene will not significantly improve normalization.

Across different tissues, *β-ACT*, *RPL7*, and *BTF3* were evaluated as the top three stable genes by Normfinder and ΔCt method. Besides, *BTF3* and *RPL7* were the most stably expressed reference genes recommended by geNorm. *SDHA* was identified as the least reference gene by geNorm, Normfinder, and ΔCt method, while it was determined as the most stable reference gene by BestKeeper (Table 2). According to the results of RefFinder, the stability ranking of the candidate reference genes from the most stable to least stable across different tissues were as follows: *β-ACT* > *RPL7* > *BTF3* > *GAPDH* > *SDHA* > *EF1α* > *28S* (Figure 3A). The results of geNorm showed that pairwise variation values were all less than 0.15 cut-off (Figure 4A). Thus, the combination of *β-ACT* and *RPL7* were the most suitable for qRT-PCR data normalization across different tissues (Table 3).

### 3.4. Expression Stability of Seven Reference Genes Across Different Temperature Treatments

For diverse temperature treatments, *EF1α* and *β-ACT* were determined to be the top two stable genes by geNorm and BestKeeper, whereas *GAPDH* and *RPL7* were recommended as the top two stable genes using Normfinder and ΔCt method. Additionally, results of four programs demonstrated that *28S* was the least stable gene (Table 2). According to the results of RefFinder, the ranking order from the most stable to least stable across different temperature treatments were as follows: *β-ACT* = *RPL7* > *EF1α* > *GAPDH* > *SDHA* > *BTF3* > *28S* (Figure 3B). The results of geNorm showed that the pairwise variation value for V_2/3_ was below 0.15 cut-off (Figure 4B). Hence, the same as different tissues, the combination of *β-ACT* and *RPL7* were the most suitable for qRT-PCR data normalization across different temperature treatments (Table 3).

### 3.5. Expression Stability of Seven Reference Genes Across Different Sexes

For different sexes, geNorm, Normfinder, and ΔCt method revealed *EF1α* and *SDHA* as the top two stable genes. However, BestKeeper predicted *GAPDH* and *RPL7* as the most stable genes. The same as different temperature treatments, *28S* was determined to be the least stable gene by all four algorithms across different sexes (Table 2). Based on the results of the RefFinder, the ranking order from the most stable to least stable across different sexes were as follows: *EF1α* > *SDHA* > *RPL7* > *GAPDH* > *β-ACT* > *BTF3* > *28S* (Figure 3C). The results of geNorm suggested that all the pairwise variation values were below 0.15 cut-off (Figure 4C). Accordingly, the combination of *EF1α* and *SDHA* were suitable for normalizing qRT-PCR data across different sexes (Table 3).

### 3.6. Validation of Recommended Reference Genes

**Figure 5:**
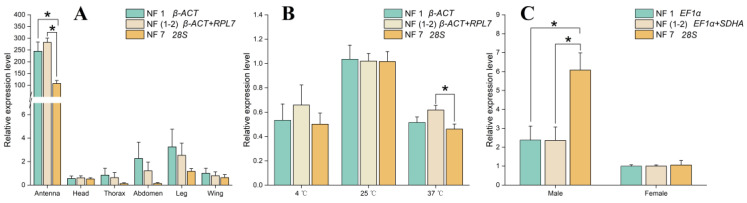
Relative expression levels of the target gene *CsacPBP1* in (**A**) Adult tissues, (**B**) Temperatures, and (**C**) Sexes were normalized by the most stable reference gene (NF1), the recommended stable reference genes (NF1-2) and least stable reference gene (NF7). The data represents the mean values ± SE. Bars represent the means and standard errors of three biological replicates. Asterisks indicate significant difference (* *p* < 0.05, student’s *t*-test). The abbreviations are listed in the description of Figure 1.

To validate the performance of recommended reference genes, the expression level of target gene *CsacPBP1* across three experimental conditions was normalized using the most stable reference gene (NF1), the combination of two most stable reference genes (NF1-2) and the least stable reference gene (NF7). Across different tissues, the expression level of *CsacPBP1* was higher in antennae than in the other five tissues, regardless of whether NF1 (*β-ACT*), NF1-2 (*β-ACT* and *RPL7*), or NF7 (*28S*) was utilized for the normalization. However, the expression level of *CsacPBP1* in antennae normalized using the NF1-2 were significantly different from using the NF7 (*p* < 0.05) (Figure 5A). For the experiment with distinct temperatures, the *CsacPBP1* expression level at 25 °C was higher than those at 4 °C and 37 °C, no matter whether NF1 (*β-ACT*), NF1-2 (*β-ACT* and *RPL7*), or NF7 (*28S*) was used as reference genes. Nevertheless, the *CsacPBP1* expression at 37 °C normalized by NF1-2 was significantly different from those calculated using NF7 (Figure 5B). *CsacPBP1* exhibited male-biased expression patterns under different sex condition, regardless of whether it was normalized by NF1 (*EF1α*), NF1-2 (*EF1α* and *SDHA*), or NF7 (*28S*). However, the expression level of *CsacPBP1* in the males using NF1-2 as reference genes were significantly different from that using the NF7 as a reference gene (*p* < 0.05) (Figure 5C).

## 4. Discussion

In this study, the stability of the expression of seven reference genes in *C. sacchariphagus* regularly used for qRT-PCR data normalization was evaluated under different experimental conditions (tissues, temperatures, and sexes). The results demonstrated that there is no reference gene that maintains a constant expression level across all conditions tested. These results coincide with those in previous studies, which reported that no universal reference gene exists that is suitable for all experimental conditions, because the expression stability of reference genes was influenced by many factors such as insect sex, tissue, life stage, temperature conditions, and so on [36,37,38,39]. Some researchers attempted to find a suitable reference gene that can express stably in various tissues of several species. The outcomes indicated that such perfect reference gene that is appropriate for all variables may not exist [21,28,40]. Therefore, it is indispensable to validate the expression steadiness of reference genes under a specific tested condition before using them to investigate target gene expressions.

To date, the fitting reference genes for many Lepidoptera species have been selected or verified under different tested conditions, including *Chilo suppressalis* [41], *Hyphantria cunea* [42], *Bombyx mori* [43], *Mythimna loreyi* [44], *Spodoptera frugipreda* [45], and *Spodoptera litura* [46]. However, the reference genes for *C. sacchariphagus* have not been screened in earlier studies. *C. sacchariphagus* is the most serious stalk borer of sugarcane because the larvae that damage sugarcanes bore into the stems, which makes the chemical control suboptimal for this pest. Moreover, frequent application of pesticides is not advisable due to their potential risks to the environment and food safety as well as pesticide resistance. Therefore, an environmental-friendly strategy for controlling *C. sacchariphagus* adults is desirable. To improve the harmless control of *C. sacchariphagus* adults, it is important to investigate the molecular basis concerning thermo-tolerance and olfaction of *C. sacchariphagus* adults. To learn more regarding the expression profiles of the genes relevant to thermo-tolerance and olfaction, the first thing we should do is the selection of suitable reference genes. In this context, the expression steadiness of seven reference genes were evaluated in this study under three distinct tested conditions.

Here, four commonly used programs (geNorm, Normfinder, Bestkeeper, and ΔCT) were utilized to evaluate the suitability of reference genes. The results reveal that no individual reference gene can achieve the top ranking across all four programs’ results across different experimental conditions, which further highlights the significance of selecting appropriate reference genes according to the specific experimental conditions in different species (Figure 3). Interestingly, geNorm, Normfinder, and ΔCT method identified a same most stable reference gene, while the Bestkeeper ranked another reference gene as the most stable reference gene. For instance, geNorm, Normfinder, and ΔCT method evaluated the *EF1α* as the top-ranking reference gene in different sexes, while the Bestkeeper ranked it as the fourth stable reference gene, with the *GAPDH* ranked as the most stable (Table 2). The identical differences between Bestkeeper and the other three programs were also found in *Chilo suppressalis* [41] and *Leptocybe invasa* [47]. These differences may be attributed to different statistical algorithms of each program. The geNorm ranks the expression stability of each reference gene by computing its expression stability value (M). The NormFinder employs a model-based strategy to assess the expression variation of each reference gene. The BestKeeper uses raw data (Ct values) and PCR amplification efficiency to create an index that indicates gene stability. The ΔCt approach relies on relative pair-wise comparisons, and the standard deviation (SD) is used to rank stability. The detail algorithms for the aforementioned four programs can be found in the documents [21,29,30,31]. Under such circumstances, RefFinder, a web-based tool that has been widely used in recent research, encompassing *Mylabris sibirica* [20], *Spodoptera frugipreda* [45] and *Spodoptera litura* [46], calculates the geometric mean of the rankings achieved by the different algorithms to address the limitations of using one single program. This enabled the creation of a consensus rating for the stability of the reference genes [32].

*β-ACT* was one of the most commonly used reference genes for qRT-PCR data normalization. Actin is the major component of microfilaments in the cytoplasm and is involved in processes such as cell motility, division, and intracellular information transmission [48]. Our results indicated that *β-ACT* was the most stable reference gene under diverse temperatures and in different tissues (Figure 3). Consistent with our results, *β-ACT* was the most stable reference gene in *Bradysia odoriphaga* under different insecticide stresses and in different sexes [39], *Anastatus japonicus* in different tissues [49], and *Hermetia illucens* across different life stages [50]. On the contrary, *β-ACT* was the least stable reference gene in *Mythimna loreyi* [44], *Spodoptera frugipreda* [45], *Hippodamia convergens* [51], and *Cimex hemipterus* [52]. In our results, *β-ACT* was not recommended as a reference gene for normalization in different sexes in *C. sacchariphagus* adults. It is generally assumed that *β-ACT* is stably expressed, for it is constitutively expressed in cells. However, the expression of *β-ACT* can be influenced by proliferation, activation, and differentiation, which explain why *β-ACT* exhibits such variability across different studies [36]. Consequently, there was no reference gene appropriate for all insects across all experimental conditions, even if it was the most commonly used reference gene for qRT-PCR data normalization.

Ribosomal proteins are foundational components of ribosomes [53]. *RPL7* was also the most stable reference gene under diverse temperatures, tied for first place with *β-ACT* (Figure 3B). Similar to our results, ribosomal proteins were evaluated as the most stable reference gene, such as in *Riptortus pedestris* under different life stages (*RPL7A*) [54], *Henosepilachna vigintioctomaculata* (*RPL6* and *RPL32*) [55] and *Phthorimaea operculella* (*RPL13*) [38] under different life stages and diverse temperatures, and *Spodoptera frugiperda* under diverse temperatures (*RPL10*) [56]. Elongation factor 1 alpha is among the most prevalent proteins and plays an important role in protein translation [26]. *EF1α* was the most stable reference gene in different sexes (Figure 3C). In the previous studies, *EF1α* exhibited a stable expression in *Tuta absoluta* exposed to 20E and under different insecticide stresses [57], *Athetis dissimilis* under different life stages, different insecticide stresses and starvation treatment [58], and *Aphidoletes aphidimyza* under sugar and starvation treatment [59].

It is worth noting that *28S* was found to be the least stable reference gene across all three experimental conditions, indicating that *28S* was not suitable for data normalization in *C. sacchariphagus* (Figure 3). However, rRNAs, including *18S* and *28S*, are thought to be the ideal reference genes for qRT-PCR data normalization as they are considered to be relatively stable across different conditions [18,26,60]. Besides, some research also showed that *28S* was evaluated as the unstable reference gene so that it was not suitable to be used for qRT-PCR data normalization under specific conditions in some insects, such as *Lymantria dispar* under different insecticide stresses and different levels of CO_2_ stress [23], *Bombyx mori* in different tissues [43], *Helicoverpa armigera* under diverse temperatures [61], and *Sitobion avenae* in population density [62]. On the other side, *28S* has been selected as the most stable reference gene, encompassing *Hippodamia convergens* and *Helicoverpa armigera* under different life stages [51,61], *Sitobion avenae* under different insecticide stresses [62], and *Acyrthosiphon pisum* in different tissues [63]. These findings once again demonstrated that even the ideal reference gene was not consistently stable across all experimental conditions. Therefore, it is essential to select corresponding reference genes for qRT-PCR data normalization according to the specific experimental conditions.

The expression of the target gene *CsacPBP1* was normalized under different experimental conditions to validate our findings. Pheromone binding proteins, which primarily exist in the sensilla trichodea, can bind to sex pheromones and play a significant role in the recognition process of sex pheromones. Our results indicated that the expression level of *CsacPBP1* was significantly different when normalized to the combination of two most stable reference genes, compared to normalized to the least stable reference gene. Thus, selecting inappropriate reference genes for data normalization may generate false positive and negative gene expression data.

## 5. Conclusions

In this study, the expression stabilities of seven candidate genes in *C. sacchariphagus* across three experimental conditions were evaluated. The findings suggested that the combinations of the two most stable reference genes were required for accurate qRT-PCR data normalization under all three experimental conditions. These combinations included *β-ACT* and *RPL7* for different tissues and diverse temperature treatments and *EF1α* and *SDHA* for different sexes. To date, this study represents the first kind in screening out suitable reference genes for gene expression analysis in *C. sacchariphagus*. Information from this study is poised to galvanize future inquiry into the gene expression of *C. sacchariphagus*, an economically important insectpest of sugarcane. For instance, the findings of this research have facilitated our future work on olfactory genes expression in *C. sacchariphagus*, which will enhance our understanding of this pest olfaction, which can serve as an important reference for developing attractants to monitor or trap *C. sacchariphagus*.

## Figures and Tables

**Table 1:** Primer information used for qRT-PCR experiment of candidate reference genes and target gene *PBP1*.

Gene Symbol	Gene Name	Primer Sequence (5′-3′)	Length (bp)	Efficiency (%)	*R* ^2^
*β-ACT*	Beta-Actin	F: CAATCCTAAAGCCAACAGA	180	95.06	0.9948
		R: GCGTAGCCCTCGTAGAT			
*GAPDH*	Glyceraldehyde-3-phosphate dehydrogenase	F: CATGCCACTACTGCTACCC	131	101.47	0.9922
		R: GGAATGACTTTGCCTACGG			
*BTF3*	Basic transcription factor 3	F: AAGAAGGTTGTTCACGCTAC	155	91.75	0.9994
		R: GCTTGTGCTTTCGGATTA			
*28S*	28S ribosomal	F: TCGCAGAATGTAGCAGGTT	129	90.02	0.9990
		R: AGCATTGATTCGGGTCCTC			
*RPL7*	Ribosomal protein L7	F: TTTTGTTATCCGTATTCGTG	131	95.61	0.9989
		R: ACAGTCGCCTTGTTGAGA			
*EF1α*	Elongation factor 1 alpha	F: GCTCTGCTCGCTTTCACC	90	107.87	0.9994
		R: TCGGGATTCACTGTATGG			
*SDHA*	Succinate dehydrogenase complex subunit A	F: AGAGGTGATAACGCACTACAA	86	105.35	0.9993
		R: CGTGAACAGAGGCACAAGA			
*PBP1*	Pheromone binding protein 1	F: CGCTGATTCGGACAC	158	99.59	0.9995
		R: TCACCTCTACACTGGGAT			

F: forward primer; R: reverse primer; *R*^2^: coefficient of correlation.

**Table 2:** Expression stability ranking of candidate reference genes under different experimental conditions.

Conditions	Reference Gens	geNorm		Normfinder		BestKeeper		ΔCT	
		Stability	Rank	Stability	Rank	Stability	Rank	Stability	Rank
Tissues	*β-ACT*	0.363	5	0.198	1	0.82	3	0.41	1
	*GAPDH*	0.254	3	0.325	4	1.14	7	0.44	4
	*BTF3*	0.151	1	0.315	3	1.10	6	0.43	3
	*28S*	0.318	4	0.335	5	1.06	5	0.46	5
	*RPL7*	0.151	1	0.286	2	1.03	4	0.42	2
	*EF1α*	0.428	6	0.406	6	0.59	2	0.49	6
	*SDHA*	0.456	7	0.460	7	0.54	1	0.53	7
Temperature	*β-ACT*	0.161	1	0.390	4	0.59	2	0.51	3
	*GAPDH*	0.380	5	0.069	1	1.04	5	0.45	1
	*BTF3*	0.477	6	0.513	6	1.36	6	0.62	6
	*28S*	0.544	7	0.650	7	1.43	7	0.71	7
	*RPL7*	0.278	3	0.179	2	0.86	4	0.45	1
	*EF1α*	0.161	1	0.437	5	0.57	1	0.54	4
	*SDHA*	0.326	4	0.386	3	0.83	3	0.54	4
Sex	*β-ACT*	0.199	5	0.274	3	0.51	5	0.45	5
	*GAPDH*	0.129	4	0.326	5	0.37	1	0.42	4
	*BTF3*	0.352	6	0.489	6	1.01	6	0.62	6
	*28S*	0.483	7	0.777	7	1.19	7	0.81	7
	*RPL7*	0.109	3	0.287	4	0.38	2	0.39	3
	*EF1α*	0.063	1	0.100	1	0.47	4	0.34	1
	*SDHA*	0.063	1	0.145	2	0.46	3	0.35	2

The rank is determined by the stability value; the lower the value, the more stable it is.

**Table 3:** Best-performing reference genes in *C.sacchariphagus* for different experimental conditions.

Conditions	Recommended Reference Genes	
Tissues	*β-ACT*	*RPL7*
Temperature	*β-ACT*	*RPL7*
Sex	*EF1α*	*SDHA*

## Data Availability

Data is contained within this article and the Supplementary Materials.

## Supplementary Materials

The following supporting information can be downloaded at: https://www.mdpi.com/article/10.3390/insects15080594/s1, Figure S1: 1% agarose gel electrophoresis experiments result of amplification products of seven reference genes; Table S1: The sequences of seven reference genes.
