# Supplementary material for: Evaluation and Validation of Reference Genes for Gene Expression Analysis Using qRT-PCR in the Sugarcane Stem Borer Chilo sacchariphagus (Lepidoptera: Pyralidae)
Source: Insects. 2024 Aug 4;15(8):594. doi: 10.3390/insects15080594 (PMC11354500; doi:10.3390/insects15080594)
Supplement: Supplementary file 1 [file insects-15-00594-s001.zip › Figure S1.pdf]

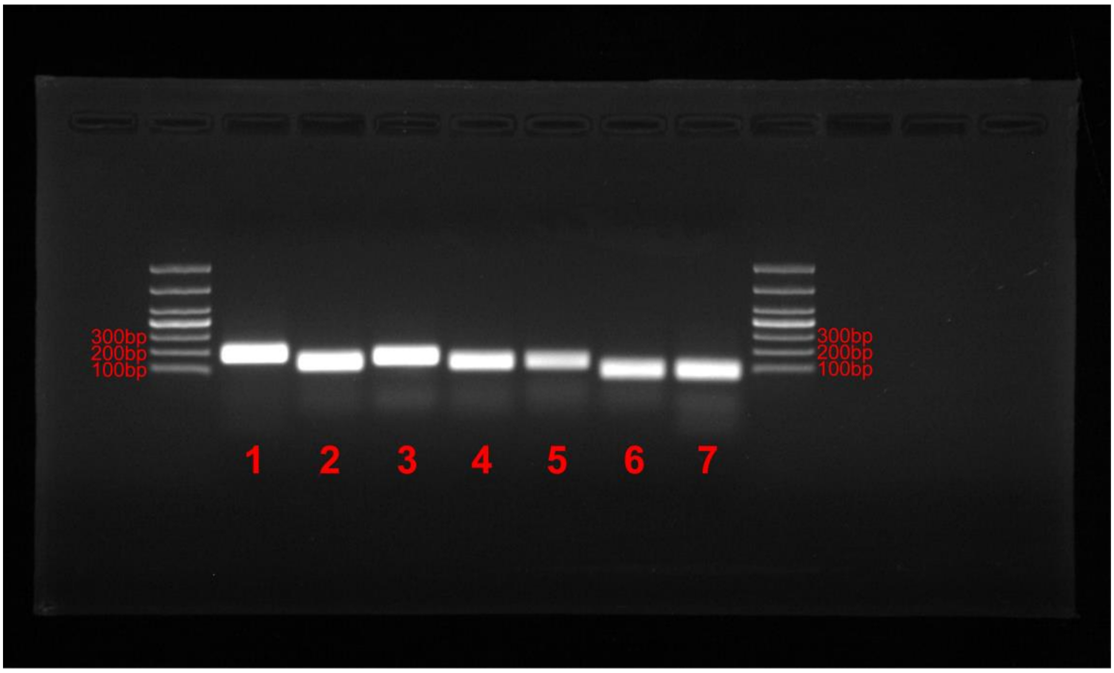

Figure S1. 1% agarose gel electrophoresis experiments result of amplification products of seven reference genes. 1: *β-ACT*; 2: *GAPDH*; 3: *BTF3*; 4: *28S*; 5: *RPL7*; 6: *EF1α*; 7: *SDHA*.
